# Improving safety claims in digital health interventions using the digital health assessment method

**DOI:** 10.1177/20552076241258756

**Published:** 2024-07-25

**Authors:** Stuart Harrison, Carsten Maple, Gregory Epiphaniou, Theodoros N Arvanitis

**Affiliations:** 1 Institute of Digital Healthcare, Warwick Manufacturing Group (WMG), 2707 University of Warwick, Coventry, UK; 2 School of Engineering, 1724 University of Birmingham, Birmingham, UK

**Keywords:** Health information technologies, device safety, medical, hazard management, medical informatics application

## Abstract

**Objective:**

Establish a relationship between digital health intervention (DHI) and health system challenges (HSCs), as defined by the World Health Organization; within the context of hazard identification (HazID), leading to safety claims. To improve the justification of safety of DHIs and provide a standardised approach to hazard assessment through common terminology, ontology and simplification of safety claims. Articulation of results, to provide guidance for health strategy and regulatory/standards-based compliance.

**Methods:**

We categorise and analyse hazards using a qualitative HazID study. This method utilises a synergy between simplicity of DHI intended use and the interaction from a multidisciplinary team (technologists and health informaticians) in the hazard analysis of the subject under assessment as an influencing factor. Although there are other methodologies available for hazard assessment. We examine the hazards identified and associated failures to articulate the improvements in the quality of safety claims.

**Results:**

Applying the method provides the hazard assessment and helps generate the assurance case. Justification of safety is made and elicits confidence in safety claim. Controls to hazards contribute to meeting the HSC.

**Conclusions:**

This method of hazard assessment, analysis and the use of ontologies (DHI & HSC) improves the justification of safety claim and evidence articulation.

## Introduction

The World Health Organization (WHO) defines digital health interventions (DHIs) as digital health functionality that enable improvements in patient safety and health strategies and address health system challenges (HSCs).^1^ DHIs provide a classification scheme or taxonomy, enabling a digital function to be aligned to HSCs, for example, the transmission of health events to specific population groups, diagnostic results, or screening services. The HSC is a health service problem or need to be addressed (e.g. lack of access to information or data, poor patient experience). The DHI is the class of technology intervention that aims to address the health service challenges. The adoption of digital tools within the health industry is hindered by the disparity between health datasets, interoperability between technology solutions and societal influences of a lack of expertise in the use of these technologies available.^2^ The pace of technology versus a perceived inflexible regulatory legislation impacted the modernisation of the health economy. The influence of digital technology users (healthcare professionals and patients) on DHI development and implementation has improved digital transformation efforts post COVID-19 pandemic, particularly in the development and implementation of DHIs.^3,4^ Post COVID-19 pandemic influences on data sharing, improvements in interoperability and data linkage have accelerated the progress being made. The regulatory challenges presented prior to the pandemic are lessening through the influences of improvements in health data access and legal reform.^3,5^ As these health industry challenges are addressed it is natural to expect that insight and influence from other industries are key. There is a greater volume of interventions available to be implemented and used within society, to meet these challenges.

Technology advances, health industry demand and post pandemic digitisation has increased the availability of DHIs. The Internet of Medical Things (IoMT) through expended network availabilities across cloud-based infrastructure enhancements and improved networks such as 5G. Big data analytics and blockchain improving health record interoperability, clinical decision-support analysis and operational support to health organisations. Artificial Intelligence, specifically improving symptom checking clinical decision-support, patient health and well-being mobile health apps. The digital health industry is predicted to grow at a compounded annual growth rate of 23.3%, from $452.79 billion in 2023 to $1965.30 billion by 2030.^6,7^

The problems of understanding the limitations of a DHI in regard to its intended use (associated safety claims) and assessing the quality of these safety claims (high-level assertions or statements of quality and safety) become a much larger scale issue. The fundamental principles of the DHI safety claim and the legislative and regulatory challenges can be addressed partly through the improvement in communication between stakeholders – regulator, health organisation, patient and health-care professionals, throughout the lifecycle of the DHI. The safety (freedom from unacceptable risk) of a DHI can be declared (as safety claim) and is supported by evidence (incorporating risk management activities performed during the lifecycle of the DHI). Risk is a combination of the probability (or likelihood) of occurrence of harm and the severity of that harm.^8,9^ Probability includes the exposure to a hazardous situation and the potential to avoid or limit the harm. Evidence aligned to safety claims may originate from the product's intended use, performance and the justification of risks presented by the implementation and use of the product through risk management methodologies. The safety claim forms part of standards and regulatory based compliance across many sectors in health software (including medical devices). The relationship between DHI and HSC, when used within the context of hazard identification (HazID), leading to safety claims, can offer insight and affirm or establish confidence that the DHIs are safe and fit for purpose.

### Safety, security and useability

The use of standards within the development of DHIs provides a consistent approach to complying with legislation. In many instances, standards are harmonised with legislation; for example, medical device legislation utilises this approach internationally for quality and risk management, design and development lifecycles.^10^ The goal of DHI manufacture and placement into the market is one of providing a product that is safe to use. This is underpinned by the security of the product (vulnerabilities in computer security as stand-alone or connected systems), effective and consistent approach to its operation within the context of intended use and the user interface.^11^

We define terms associated with safety and risk assessment within this research to provide a common understanding in support of the contributing factors and the methodology promoted (Table 1).

**Table 1.** Terms and definitions associated with risk assessment of DHIs.

Safety	Freedom from unacceptable risk^12^
security	Resistance to intentional, unauthorised act(s) designed to cause harm or damage to a system^13^
Useability	Human factors engineering application of knowledge about human behaviour, abilities, limitations and other characteristics to the design of medical devices (including software), systems and tasks to achieve adequate useability^14^ Achieving adequate USEABILITY can result in acceptable RISK related to use.
Risk	combination of the probability of occurrence of harm and the severity of that harm^12^
Harm	injury or damage to the health of people, or damage to property or the environment^12^
Hazard	potential source of harm^12^
Hazardous situation	circumstance in which people, property, or the environment is/are exposed to one or more hazards^12^

Security is one part of the overall safety claim, as are other domains defined in standards and legislation relating to health software and medical device development, implementation and use. The definition of ‘safety’ in ISO 14971 and underlying ISO and IEC guidance, safety incorporates harm that affects health, property and environment. Safety is not just related to the health of patients, that definition of harm also includes any adverse outcome of malicious attacks we assess when we discuss security. The risk management standard ISO 14971 requires the examination of harm even if the device is not used according to its intended purpose, in case of foreseeable misuse. It is required to consider the environment the device will be used in, in this regard. So, malicious attacks are not excluded. Security vulnerabilities can compromise operations of DHIs, accessibility, integrity and availability.^15^

**Figure 1.**
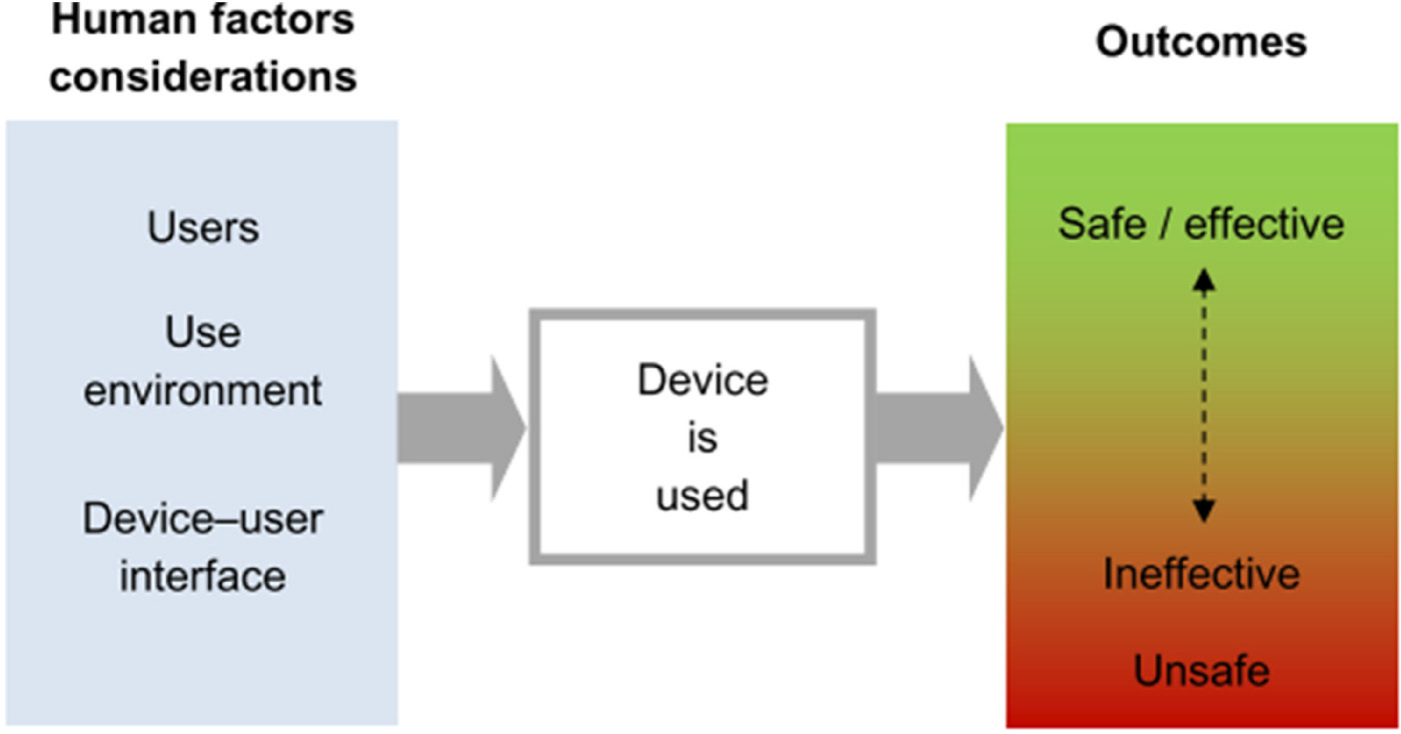
Human factors affect the outcomes of using a medical device.^17^

An important subject or domain that influences the effectiveness of DHIs and the associated safety claim is the science of human factors and useability (Figure 1). Human factors refer to the interaction of people with the systems surrounding them, including the technology they use. The development or design of DHIs has an essential part to play with the safe implementation and use of these technologies. As with the placing of a product in the market place or industry (i.e., digital health) by a manufacturer, the intended purpose of the DHI is critical. This is explored in more detail relating to the methodology used in this research. Intended purpose, patient cohort, user competency (including cognitive behaviour) and environment all play a part in this domain. Human factors within the Digital Health industry and healthcare in general has learned from safety critical industries and has become an essential domain within the development of DHIs, underpinning safety claims. As such within the industry we encompass terms such as ergonomics and useability. For the purposes of this research, we use the term useability engineering. We define the term Ergonomics (or human factors) as the scientific discipline concerned with the understanding of interactions among humans and other elements of a system and the profession that applies theory, principles, data and methods to design in order to optimise human well-being and overall system performance.^16^

Useability engineering within the design and development of DHI products relate to the safety claims declared. The application of risk management focusses on design flaws, manufacturing failures, electrical issues and the correct or incorrect use of the product (foreseeable misuse). Risk control measures are applied during the design, development and production part of the product lifecycle^14,18^ The inclusion of useability into the risk assessment of a DHI requires domain specific goals that must align with the safety claim – objectives, roles and target performance indicators.^19^

### Application of risk management

We postulate the application of risk management methodologies to identify hazards, can be linked to DHI classes and HSC categories thereby improving safety justifications (or claims) made. There are other influencing factors across the domains of Information Governance, Privacy, Information Security, Cyber Security and Useability; our objective is improving quality through the utilisation of these domains during risk assessment, from common terms and structured statements concerning hazard assessment. We acknowledge the influences on DHI safety through the implementation and use, as well as the development lifecycle; the focus of this research is to establish a relationship between DHI and HSCs (HSCs), as defined by the WHO; within the context of HazID, leading to safety claims. To improve the justification of safety of DHIs and provide a standardised approach to hazard assessment through common terminology, ontology and simplification of safety claims. Articulation of results, to provide guidance for health strategy and regulatory/standards-based compliance.

We categorise and analyse hazards using a qualitative HazID study. This method utilises a synergy between simplicity of DHI intended use and the interaction from a multidisciplinary team (technologists and health informaticians) in the hazard analysis of the subject under assessment as an influencing factor.^20^ Although there are other methodologies available for hazard assessment, the method chosen enables examination of the research objective rather than the effectiveness of risk assessment methodology. In safety critical industries, multiple methods of risk assessment are utilised to establish both proactive and reactive risk treatment plans.^21^ This also includes the utilisation of libraries of methodologies and applicability to system specifications. The steps presented and associated assessments aim to contribute (qualitatively) via the application of a safety analysis and assessment methodology to generate improved safety claims, standardise terms used and correlate towards classifications of DHI and HSCs. We aim to present a direct correlation between HSC, the safety justification and building of structured safety claims using taxonomies through safety engineering methodologies. The benefits of HazID align with a more structured data driven approach where the utilisation or standard terms and categories of risk move towards a more effective assessment.^22,23^ HazID – hazard identification, is utilised in safety critical industries (including digital health) often requires that all hazards with the potential to cause harm are identified. The methodology provides a structured approach to HazID and the assessment of likelihood and severity.^24^

The health industry is influenced by safety critical industry practices where patient safety is paramount and the foundations of safety engineering concepts and methods can be seen to improve quality and safety.^25,26^ However, safety claims about such interventions vary in quality. Quality improvement concerns the consistency of terminology, repeatability of safety analysis and assessments made and standardisation in the description of safety claims. These quality issues impact the hazard assessments performed and subsequent regulatory evidence supporting compliance.^2,27^ The communication of the safety claims between technical and clinical experts and other stakeholders, such as users of DHIs is open to confusion, lack of understanding and limited expertise in safety engineering concepts or regulatory requirements.^25,28,29^ The claims made for DHIs often support regulatory requirements that enable a product to be placed into the market. This aligns with the objectives of good manufacturing practise, health software quality development, medical device manufacturer and the supporting standards and regulations that align to best practise.

The lack of the adoption of the fundamental concepts of clinical risk management (CRM) and safety methods, within health informatics, demonstrates safety's limited influence in the development of digital health technologies.^30,31^ It has been shown that the foundations of safety engineering concepts and methods can improve quality and safety.

‘Adopting a systematic approach to risk management, building on best practice in system safety, has been beneficial. Much of the benefit has been realised due to the close engagement by the clinicians. However, despite such progress, more work remains in order to mature current safety assurance practices and improve organisational support’.^25^

The impact of DHIs on the safety of patients and potential harm exercised by the unsafe actions of clinical users, is not documented openly.^31–33^ Evidence suggests a lack of rigor within the industry, where strategies for innovation to improve clinical outcomes and advance health using new technologies, overlook the principles of safety.^25,28,29^ The exponential growth, diversity of DHIs and associated regulatory position are the biggest challenges to the industry. Policy makers, manufacturers, health organisations and digital technology users (healthcare professionals and patients) have different understandings and objectives of DHIs.^30,34,35^ These strategies often, see rigor as a barrier not an enabler to innovation.^29,36,37^

In contrast to the digital healthcare industry, traditional safety critical engineering industries have the capability of in-depth analysis and assessment, while they have been established over decades. Traditional safety critical industries integrate quality, benefits and safety objectives in structured and formal methodologies that are more amenable to innovation than the healthcare industry currently exhibits.^38^ This work aims to indicate that the domain of safety can be correlated, using a structured methodology to improve the communication of safety claims of DHIs.

### Digital health interventions

The benefit and contribution to the communication between stakeholders, for safety claims aligned with the classifications of DHIs is presented in this paper. The safety of a digital health product can be declared (safety claim) and supported by evidence from the intended use of the product, performance and the justification of risks presented by the implementation and use of the product through risk management methodologies (safety case or assurance case).^11,39^ We introduce the term CRM in a digital context to focus the risk management methodology on digital clinical safety. CRM is the systematic application of management policies, procedures and practices to the tasks of analysing, evaluating and controlling clinical risk. The safety claim forms part of standards and regulatory based compliance^40^ in this regard.

The WHO classification of DHIs,^1^ and their relationship to HSCs, provides us with an opportunity to establish or affirm safety claims by the application of safety analysis methods. HSC is a health service problem or need to be addressed (e.g. lack of access to information or data, poor patient experience). DHI is the class of technology intervention that aims to address the service problem (HSC). This can offer insight and establish confidence that the DHIs are safe and fit for purpose by applying safety methods. The WHO classification promotes an accessible and bridging language between technical and clinical experts aimed at simplifying dialogue and aiding digital health implementation. The classification represents discrete functionality of DHI, to achieve health sector objectives and meet the HSC. This is aimed at commissioners of digital services. By the application and use of these classifications and HSCs, we are able to identify hazards and construct a safety claim and justification. This, in turn, will bridge the understanding of the health delivery organisation and manufacturer CRM processes by way of guidance for each DHI classification.

The quality of design and development, implementation and the use of DHIs are dependent on the rigour applied from the initial concept or innovative idea to minimum viable product or prototype development and minimum marketable product. The use of relevant standards and industry guidance provides a manufacturer greater opportunity to demonstrate effectiveness and meet health sector challenges and user needs.^37,41,42^ The use of taxonomies, synonyms and ontologies is an established method of grouping common themes to assist in resolving interpretational issues within the field of health informatics.^43,44^

The Digital Health Assessment Methodology (DHAM) provides informative guidance and insight to both technical and clinical experts. This will inform the benefits of effective safety claims linked to DHI classifications and HSCs. The framework for synthesis of safety justification,^44^ enables the establishment of DHI safety related justifications within the method developed. The safety justifications are created in such a format that we can group the terms used. These taxonomies will allow for the grouping, classifying, grading, ranking, sorting and stratification of the results. The grouping further improves the communication and guidance of safety claims and relate to the HSCs faced.

HSCs provide synergy between intended operational use and the challenge faced in the health system. It is purposeful for examining the relationship between HSC, Hazard, Effect and overall DHI contribution, which is key to establishing safety claims. The HSC also provides a consistent terminology and phrasing that is less open to misinterpretation. This helps to establish consistency and focus to the hazard assessment.^45^

### Safety engineering and preliminary hazard analysis

DHIs, health software (including medical devices), are safety critical systems presenting immediate patient safety risks during normal and abnormal operating conditions to the user. The foundations of safety engineering concepts and methods are well known in the improvement of quality and safety.^25^ The relationship between a defined hazard or hazardous situation and the effect of the hazard will be assessed using this method. We postulate that the use of safety engineering methods, HazID using a defined method, enables a higher quality preliminary hazard analysis to be performed. This preliminary hazard assessment methodology is applicable to CRM requirements within medical device regulatory standards.^11,39,46,47^ We use a qualitative HazID study due to the cooperation between simplicity of understanding, the use of multidisciplinary teams and the reliance on the experience of the team in the subject under assessment. Although there are many methods available this has been selected due to the preliminary nature of assessment in the overall lifecycle and the objectives of this study (i.e., improvements in the articulation of safety claims).

### Digital health interventions and health domains

The classification scheme utilises the term System Category to represent diverse types of Information and Communication Technologies (ICT). The original objective of the WHO classification scheme was to address the need for a shared language for health researchers to enable improvements in the synthesis of evidence for innovation across a complex healthcare landscape.^48,49^ The DHIs used in the following method cover a common fixed scope of health domains (Table 2), to provide a tenable boundary for analysis and results. Further discussion will acknowledge those DHIs that fall into the category of self-care.

**Table 2.** Definitions of included DHIs.^49^

Digital health intervention	Definition	Synonyms and other descriptors
BIRTH NOTIFICATION	Digital approaches to support the notification of births, to trigger the subsequent steps of birth registration and certification and to compile vital statistics	Birth event alerts Enabling healthcare workers and community to transmit alerts/ notifications when a birth has occurred
DEATH NOTIFICATION	Digital approaches to support the notification of deaths, to trigger the subsequent steps of death registration and certification and to compile vital statistics, including cause-of-death information	Death surveillance Death event alert Enabling healthcare workers and communities to transmit alerts/ notifications when a death has occurred
STOCK NOTIFICATION AND COMMODITY MANAGEMENT	Digital approaches for monitoring and reporting stock levels and consumption and distribution of medical commodities. This can include the use of communication systems (e.g. SMS) and data dashboards to manage and report on supply levels of medical commodities	Stock-out prevention and monitoring Alerts and notifications of stock levels Restocking coordination Logistics management and coordination
CLIENT-TO-PROVIDER TELEMEDICINE	Provision of health services at a distance; delivery of health services where clients/patients and healthcare workers are separated by distance	Consultations between remote client/ patient and healthcare worker Clients/patients transmit medical data (e.g. images, notes and videos) to healthcare worker
PROVIDER-TO-PROVIDER TELEMEDICINE	Provision of health- services at a distance; delivery of health services where two or more healthcare workers are separated by distance	Consultations for case management between healthcare workers Consulting with other healthcare workers, particularly specialists, for patient case management and second opinion
TARGETED CLIENT COMMUNICATION (TARGETED COMMUNICATION TO INDIVIDUALS)	Transmission of customised health information for different audience segments (often based on health status or demographic categories). Targeted client communication may include: transmission of health-event alerts to a specified population group. transmission of health information based on health status or demographics. alerts and reminders to clients. transmission of diagnostic results (or of the availability of results).	Notifications and reminders for appointments, medication adherence, or follow-up services Health education, behaviour change communication, health promotion communication based on a known client's health status or clinical history Alerts for preventive services and wellness
HEALTHCARE WORKER DECISION SUPPORT	Digitised job aids that combine an individual's health information with the healthcare worker's knowledge and clinical protocols to assist healthcare workers in making diagnosis and treatment decisions	Clinical decision-support systems (CDSS) Job aid and assessment tools to support service delivery, may or may not be linked to a digital health record Algorithms to support service delivery according to care plans and guidelines
TRACKING OF PATIENTS’/CLIENTS’ HEALTH STATUS AND SERVICES (DIGITAL TRACKING)	Digitised record used by healthcare workers to capture and store health information on clients/patients in order to follow-up on their health status and services received. This may include digital service records, digital forms of paper-based registers for longitudinal health programmes and case management logs within specific target populations, including migrant populations.	Digital versions of paper-based registers for specific health domains Digitised registers for longitudinal health programmes including tracking of migrant populations’ benefits and health status Case management logs within specific target populations, including migrant population
PROVISION OF EDUCATIONAL AND TRAINING CONTENT TO HEALTHCARE WORKERS	The management and provision of education and training content in digital form for health professionals. In contrast to decision support, mLearning does not need to be used at the point of care.	mLearning, eLearning, virtual learning educational videos, multimedia learning and access to clinical guidance for training reinforcement

‘The World Health Organization (WHO) defines self-care as the ability of individuals, families and communities to promote health, prevent disease, maintain health and to cope with illness and disability with or without the support of a healthcare worker’.^50^

The classification scheme also draws relevance from the international standards community.

‘provides a guide to best practice business requirements and principles for planning the use of information and communications technology (ICT) to support the development, coordination and delivery of healthcare services by countries and subordinate health authorities within a country’.^51^

Aligning to the need to support a growing health information system ecosystem and infostructure, these strategies follow the common objectives of providing improvements in communication between stakeholders and removing barriers to standardisation, quality and safe implementation goals. We provide a summary of DHIs to enable further understanding (Table 3).

**Table 3.** Included digital health interventions.^48^

1.0 Clients					
1.1	Targeted Client Communication	1.3	Client to client	1.6	On-demand information services to clients
1.1.1	Transmit health event alerts to specific population group(s)	1.3.1	Peer group for clients	1.6.1	Client look-up of health information
1.1.2	Transmit targeted health information to client(s) based on health status or demographics	1.4	Personal health tracking	1.7	Client financial transactions
1.1.3	Transmit targeted alerts and reminders to client(s)	1.4.1	Access by client to own medical records	1.7.1	Transmit or manage out of pocket payments by client(s)
1.1.4	Transmit diagnostics result, or availability of result, to client(s)	1.4.2	Self-monitoring of health or diagnostic data by client	1.7.2	Transmit or manage vouchers to client(s) for health services
1.2	Untargeted client communication	1.4.3	Active data capture/ documentation by client	1.7.3	Transmit or manage incentives to client(s) for health services
1.2.1	Transmit untargeted health information to an undefined population	1.5	Citizen-based reporting		
1.2.2	Transmit untargeted health event alerts to undefined group	1.5.1	Reporting of health system feedback by clients		
		1.5.2	Reporting of public health events by clients		
2.0 Healthcare providers					
2.1	Client identification and registration	2.4	Telemedicine	2.7	Healthcare worker activity planning and scheduling
2.1.1	Verify client unique identity	2.4.1	Consultations between remote client and healthcare provider	2.7.1	Identify client(s) in need of services
2.1.2	Enrol client for health services/clinical care plan	2.4.2	Remote monitoring of client health or diagnostic data by healthcare provider	2.7.2	Schedule healthcare provider's activities
2.2	Client health records	2.4.3	Transmission of medical data to healthcare provider	2.8	Healthcare provider training
2.2.1	Longitudinal tracking of clients’ health status and services	2.4.4	Consultations for case	2.8.1	Provide training content to healthcare provider(s)
2.2.2	Manage client's structured clinical records	2.5	Healthcare provider communication	2.8.2	Assess capacity of healthcare provider(s)
2.2.3	Manage client's unstructured clinical records	2.5.1	Communication from healthcare provider(s) to supervisor	2.9	Prescription and medication management
2.2.4	Routine health indicator data collection and management	2.5.2	Communication and	2.9.1	Transmit or track prescription orders
2.3	Healthcare provider decision support	2.5.3	performance feedback to healthcare provider(s)	2.9.2	Track client's medication consumption
2.3.1	Provide prompts and alerts based according to protocol	2.5.4	Transmit routine news and workflow notifications to healthcare provider(s)	2.9.3	Report adverse drug events
2.3.2	Provide checklist according to protocol	2.5.5	Transmit non-routine health event alerts to healthcare provider(s)	2.10	Laboratory and Diagnostics Imaging Management
2.3.3	Screen clients by risk or other health status	2.6	Referral coordination	2.10.1	Transmit diagnostic result to healthcare provider
		2.6.1	Coordinate emergency response and transport	2.10.2	Transmit and track diagnostic orders
		2.6.2	Manage referrals between points of service within health sector	2.10.3	Capture diagnostic results from digital devices
		2.6.3	Manage referrals between health and other sectors	2.10.4	Track biological specimens
3.0 Health System Managers					
3.1	Human resource management	3.3	Public health event notification	3.5	Health financing
3.1.1	List health workforce cadres and related identification information	3.3.1	Notification of public health events from point of diagnosis	3.5.1	Register and verify client insurance membership
3.1.2	Monitor performance of healthcare provider(s)	3.4	Civil registration and vital statistic	3.5.2	Track insurance billing and claims submission
3.1.3	Manage certification/ registration of healthcare provider(s)	3.4.1	Notify birth event	3.5.3	Track and manage insurance reimbursement
3.1.4	Record training credentials of healthcare provider(s)	3.4.2	Register birth event	3.5.4	Transmit routine payroll payment to healthcare provider(s)
3.2	Supply chain management	3.4.3	Certify birth event	3.5.5	Transmit or manage incentives to healthcare
3.2.1	Manage inventory and distribution of health commodities	3.4.4	Notify death event	3.5.6	Manage budget and expenditures
3.2.2	Notify stock levels of health commodities	3.4.5	Register death event	3.6	Equipment and asset management
3.2.3	Monitor cold-chain sensitive commodities	3.4.6	Certify death event	3.6.1	Monitor status of health equipment
3.2.4	Register licensed drugs and health commodities			3.6.2	Track regulation and licensing of medical equipment
3.2.5	Manage procurement of commodities			3.7	Facility management
3.2.6	Report counterfeit or substandard drugs by clients			3.7.1	List health facilities and related information
				3.7.2	Assess health facilities
4.0 Data services					
4.1	Data collection, management and use	4.2	Data coding	4.3	Location mapping
4.1.1	Non-routine data collection and management	4.2.1	Parse unstructured data into structured data	4.3.1	Map location of health facilities/structures
4.1.2	Data storage and aggregation	4.2.2	Merge, de-duplicate and curate coded datasets or terminologies	4.3.2	Map location of health events
4.1.3	Data synthesis and visualisation	4.2.3	Classify disease codes or cause of mortality	4.3.4	Map location of clients and households
4.1.4	Automated analysis of data to generate new information or predictions on future events			4.4	Data exchange and interoperability
				4.4.1	Data exchange across systems

### Method – DHI hazard assessment method (DHAM)

**Figure 2.**
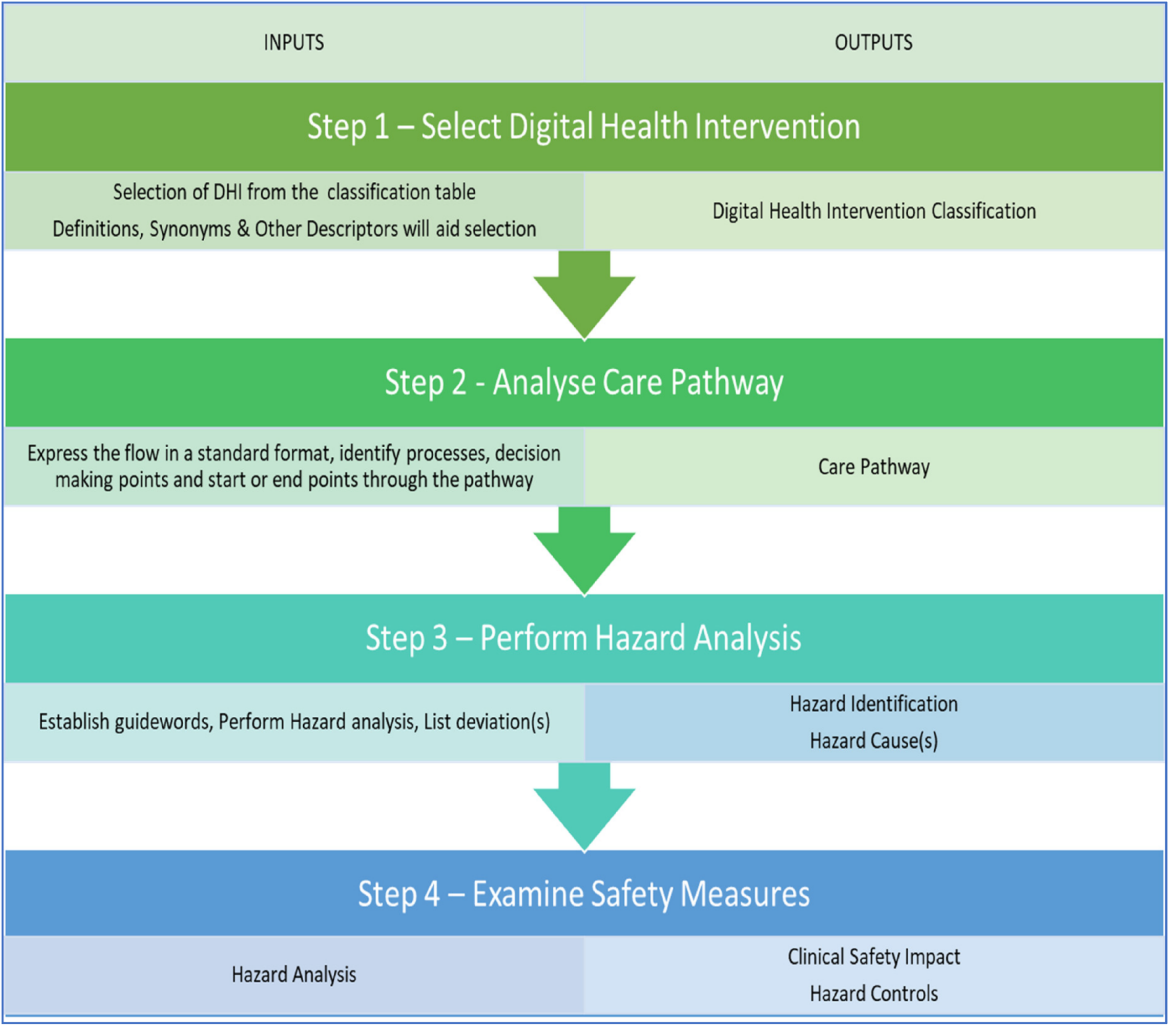
DHI Hazard Assessment Method (DHAM).

**Figure 3.**
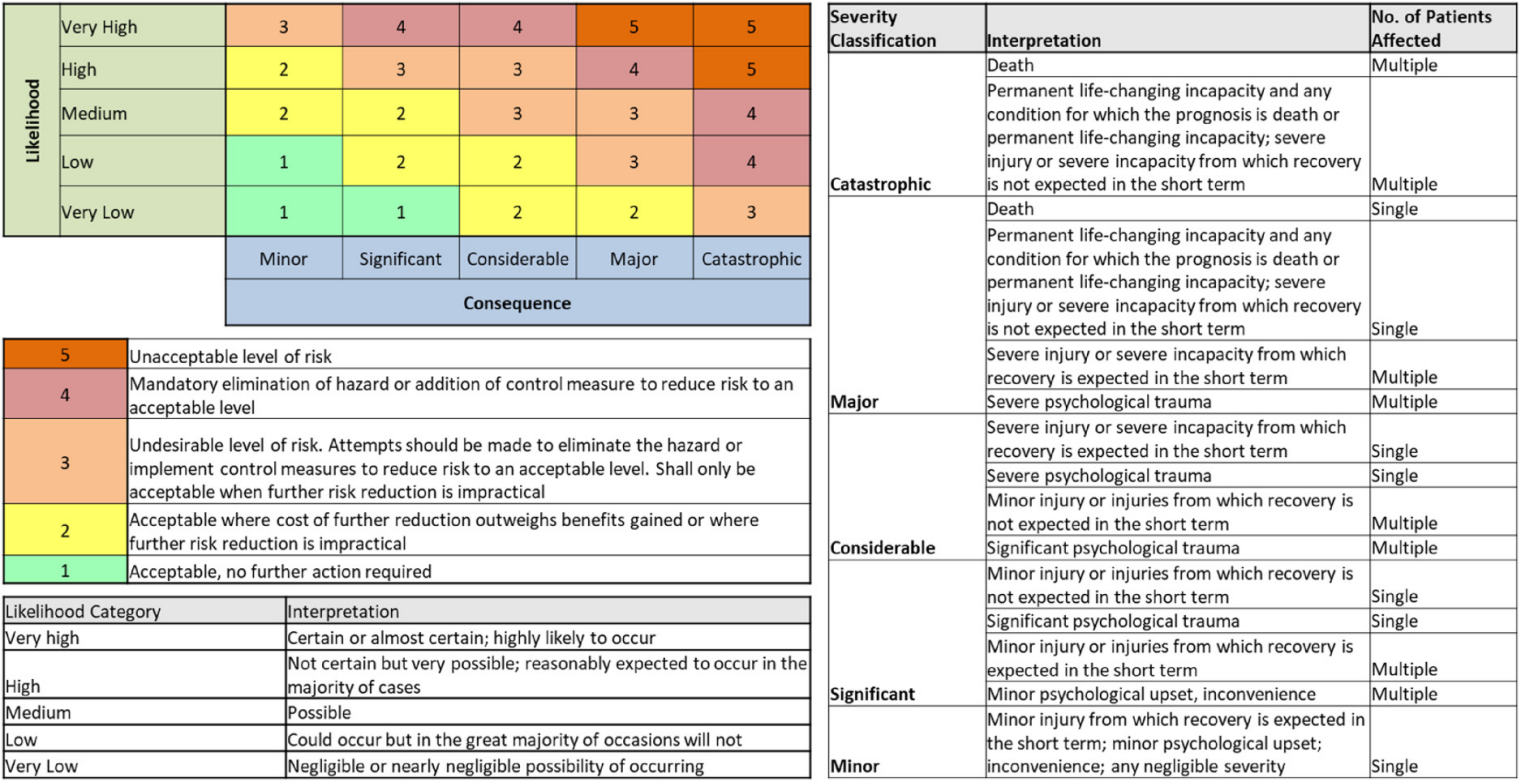
Risk matrix.

The DHI Hazard Assessment Method (DHAM) has four steps in which the DHI can be classified (Figure 2), analysed and resultant safety measures be established (Figure 3). The DHI classification scheme was used to generate guidance on the effectiveness for DHIs.

By utilising the DHI classes we categorise and analyse hazards relating to patient safety in the implementation and use of the DHI function. The safety analysis process incorporates a category of operational and deviation-based risk assessment processes – HazID (Hazard identification),^20^ Hazard and Operability Studies (HAZOP),^44^ Failure Modes and Effects Analysis (FMEA) and Functional Hazard Analysis.^2^ Completed at the early stages of development lifecycles, these methods have the advantage of highlighting risk to multidisciplinary stakeholders with a common level of understanding. A precursor to using this method may be fault tree analysis or code reviews, which are sub-system level and dependant on product experts and technologists. We have also stated that mixed methods of risk assessment performed during the DHI lifecycle are beneficial and this method allows for the constructive interaction of methods utilised.

We perform an initial HazID on a typical DHI and provide a rationale for the justification of DHIs. This four-step method was designed and applied to typical examples of Digital Health services or implementations in healthcare. We apply the method and derive safety justifications to DHIs. Examination of the hazards identified and associated failures are used to articulate potential improvements in the quality of safety claims.

### Step one – select the DHI

Select the DHI that best describes the digital health technology from Table 3. This provides an input to the method (Figure 3) and provides the initial DHI classification and ongoing study case for assessment.

Select DHI is completed using the established DHIs (Table 3). These predetermined taxonomies, from mobile applications and more traditional digital health technology solutions, ensure the DHI, definition, synonyms and other descriptors are meaningful and consistent. The objective being to align the digital health technology to the DHI class for further analysis.

### Step two – analyse the care pathway

Document the care pathway for the DHI. This pathway should allow for the examination and assessment of potential deviations from the intended or desired process flow. Potential deviations can consider omissions, early, mistake, wrong and other guidewords.^44,52^ Ensure each DHI example has the required minimum evidence for analysis and assessment – workflow, explanation of intended use, operation and clinical speciality. Noting importantly that we utilise the definition of care pathway published in literature – ‘care pathways as a method for patient care management of a well-defined group of patients during a well-defined period of time’.^51^ The care pathway must demonstrate the key components or elements of the patient flow or care journey. Care must be taken to express the flow in a standard format, identify processes, decision-making points and start or end points through the pathway, e.g. a flowchart.

Documenting this way allows us to convey the treatment of the patient in steps, allowing us to analyse care pathway deviations from these steps that may become hazards. Clinical, care, or patient pathways and other terms are used that attempt to define the patient journey (end to end or partial). We use the term care pathway and align to best practise as defined in clinical professions, international standards and health technology guidance.

### Step three – perform hazard identification

The formal methodology chosen for hazard analysis must enable an assessment to be performed such that the focus on the care pathway, is independent of technology beyond the classification of DHI. This allows us to perform HazID at a more operational assessment level. The objective of step three will aid in bridging the communication barrier between clinicians and technologists. The HazID methodology^44^ enables us to assess credible failures by examination of the use of the DHI and the deviation or divergence from that use.

The inclusion of HSCs provides constructive interaction between intended operational use and the challenge faced in the health system when a DHI does not operate as intended. We use this to highlight the relationship between hazard and defined HSCs.

These can be found summarised in Table 4.

**Table 4.** Health system challenges.

Health system challenges		
1 – Information	3 – Quality	6 – Efficiency
1.1 – Lack of population denominator	3.1 – Poor patient experience	6.1 – Inadequate workflow management
1.2 – Delayed reporting of events	3.2 – Insufficient healthcare worker competence	6.2 – Lack of or inappropriate referrals
1.3 – Lack of quality/ reliable data	3.3 – Manage inventory and distribution of health commodities	6.3 – Poor planning and coordination
1.4 – Communication roadblocks	3.4 – Low-quality health commodities	6.4 – Delayed provision of care
1.5 – Lack of access to information or data	3.5 – Low healthcare worker motivation	6.5 – Inadequate access to transportation
1.6 – Insufficient utilisation of data and information	3.6 – Insufficient continuity of care	7 – Cost
1.7 – Lack of unique identifier	3.7 – Inadequate supportive supervision	7.1 – High cost of manual processes
	4 – Acceptability	7.2 – Lack of effective resource allocation
2 – Availability	4.1 – Lack of alignment with local norms	7.3 – Client-side expenses
2.1 – Insufficient supply of commodities	4.2 – Programmes which do not address individual beliefs	7.4 – Lack of coordinated payer mechanism
2.2 – Insufficient supply of services	5 – Utilisation	8 – Accountability
2.3 – Insufficient supply of equipment	5.1 – Low demand for services	8.1 – Insufficient patient engagement
2.4 – Insufficient supply of qualified healthcare workers	5.2 – Geographic inaccessibility	8.2 – Unaware of service entitlement
	5.3 – Low adherence to treatments	8.3 – Absence of community feedback mechanisms
	5.4 – Loss to follow-up	8.4 – Lack of transparency in commodity transactions
		8.5 – Poor accountability between the levels of the health sector
		8.6 – Inadequate understanding of beneficiary populations

### Step four – examination of safety significance & control – hazard analysis

**Figure 4.**
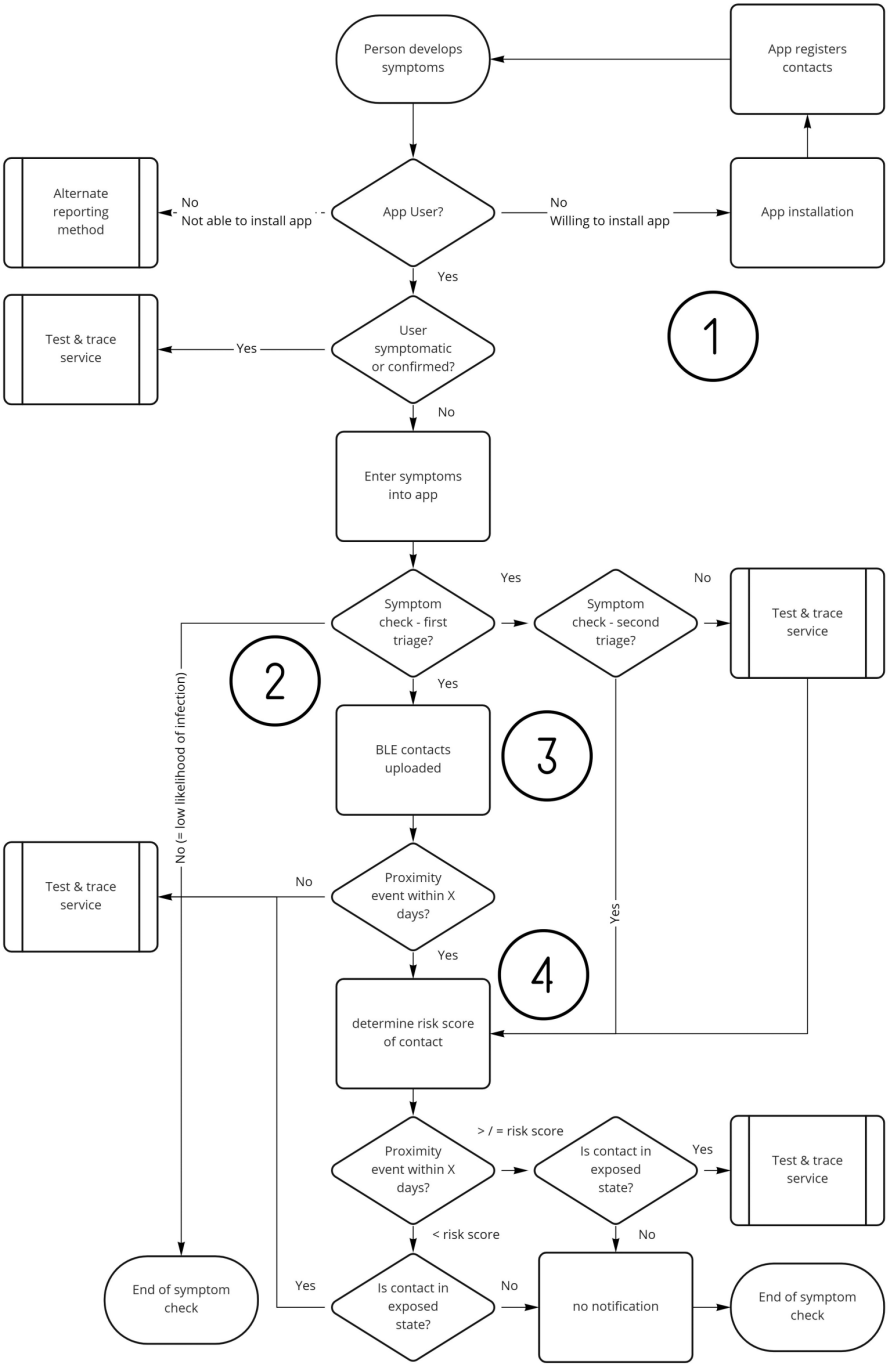
COVID-19 care pathway (flowchart) including hazard identification.

Examine safety significance & control of each hazard and *identify safety controls*. The application of CRM (HazID) forms the HazID. Examination of the DHI hazard(s) using likelihood (L) and severity (S) to derive a consequence (safety significance), completes the preliminary hazard analysis (derived from Figure 3). Examine safety significance & Identify safety controls is where CRM methods are used – hazard analysis. An examination of DHI hazards is completed using likelihood or probability of occurrence of harm and consequence to derive a severity level.^38,39^ The risk matrices (Figure 4) is a typical 5 × 5 matrices used in risk management of health software and medical devices.^38^

Identify safety controls or safeguards that enable mitigation of hazards identified. The identification of controls is performed by suitably experienced and competent personnel. The expected outcome of this stage will be identification of hazards that correlate to HSCs that form the important connection between DHI implementation and satisfying an outcome based on the HSC. It is expected that this will enable clarification of safety features for the DHI to be effective in this regard and achieve its intended purpose.

Examination of the hazard analysis performed must ensure that terminology used is unambiguous and describes the defined term without risk of miscommunicating statements. Careful reflection on the definitions of Hazard, Hazardous situation; should ensure the context is maintained (consistency and repeatable). Familiarity with the DHI is key in this regard so that explanations of events can be further analysed for grouping or consolidating causes. Finally, HSCs used together with the patient safety impact ensures the terminology is standardised.

## Results – DHI implementation using the DHAM method

### Example 1 – UK NHS COVID-19 App

The UK NHS COVID-19 app^53^ provides contact tracing functionality, a high-level symptom checking and alerting functionality. This example has been simplified from publicly available information, with the test and trace system performed by a separate government health organisation not considered within scope of this example (due to complexity).

### Step one – establish the digital health intervention functional description (DHI selection)

Select the functional description from Table 2 that represents the DHI, including the definition, synonyms and other descriptors. Table 5 summarises the functional description for this example DHI.

**Table 5.** DHI functional description.

Digital health intervention	Targeted communication to individuals
Definition	Synonyms and other descriptors
Transmission of customised health information for different audience segments (often based on health status or demographic categories). Targeted client communication includes: the transmission of health-event alerts. the transmission of health information based on health status or demographics. Alerts and reminders. Transmission of diagnostic results (or of the availability of results).	Notifications and reminders for appointments and follow-up services Health education, behaviour change communication, health promotion communication based on a known client's health status and clinical history Alerts for preventive services and wellness

### Step two – analyse the DHI-enabled care pathway

**Figure 5.**
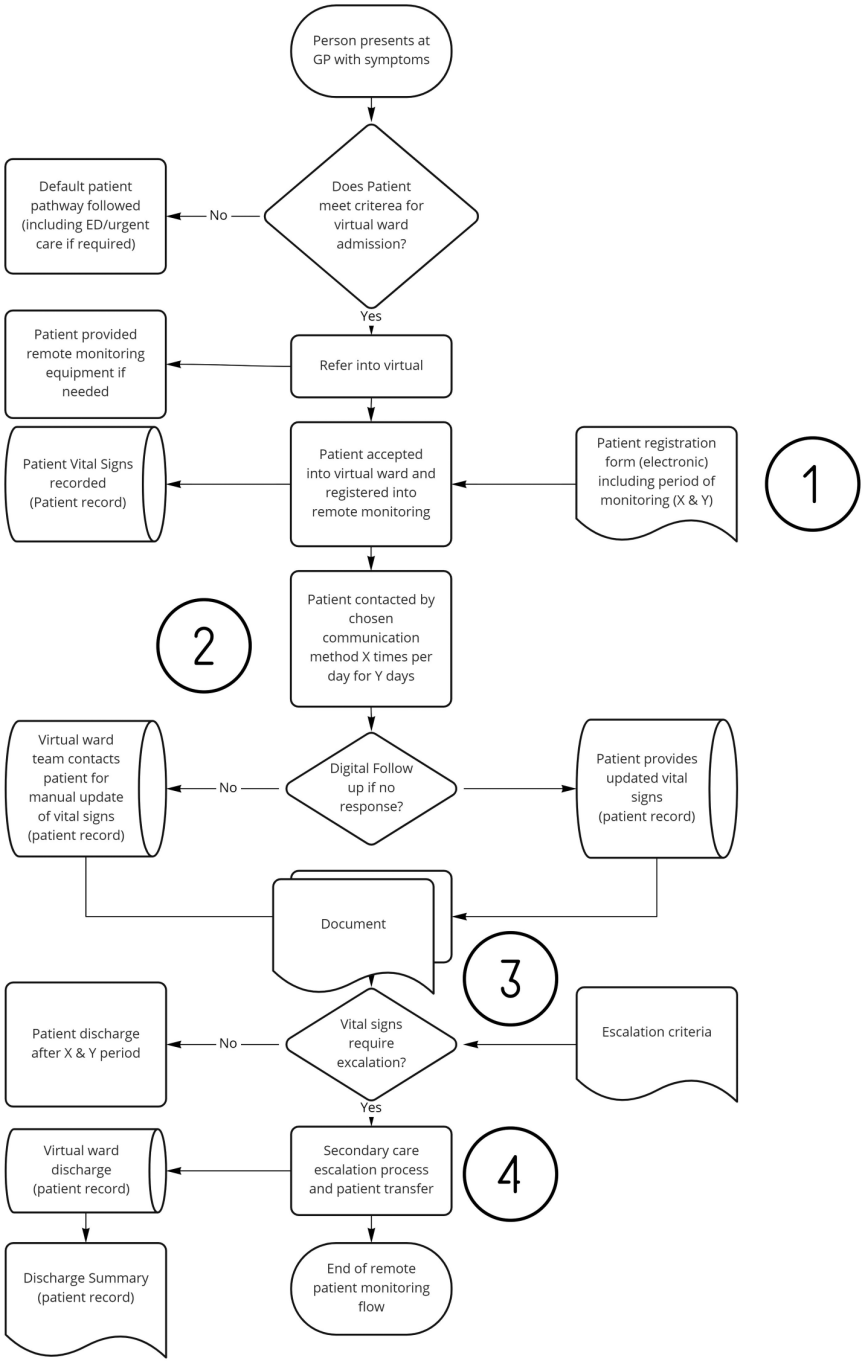
Remote patient monitoring flowchart.

Document the care pathway that the DHI is used (Figure 5). Ensure each DHI example has the required minimum evidence for analysis and assessment – product name, workflow (flowchart), explanation of intended use, technical operation and features or functions (clinical speciality). Table 6 provides the summary for this example DHI.

**Table 6.** DHI product name and technical operation summary evidence.

Product name	COVID-19 App ^53^
User Type or Role	Anyone aged 16 or over who lives in England or Wales can use the NHS COVID-19 app.
DHI technical operation	The DHI is an app running on proven software developed by Apple and Google, designed so that nobody will know who or where you are. And you can delete your data, or the app, at any time.
DHI features or functions	Trace: find out when you've been near other app users who have tested positive for coronavirus. Alert: lets you know the level of coronavirus risk in your postcode district. Check-in: get alerted if you've visited a venue where you may have come into contact with coronavirus, using a simple QR code scanner. No more form filling. Symptoms: check if you have coronavirus symptoms and see if you need to order a test. Test: helps you order a test if you need to: Isolate: keep track of your self-isolation countdown and access relevant advice.
Other Information	The app is available in multiple following languages: English, Welsh, Bengali, Urdu, Gujarati, Punjabi (Gurmukhi script), Chinese (simplified), Romanian, Turkish, Arabic (modern standard), Polish and Somali.
Care Pathway	See Figure 5

### Step three – perform hazard analysis

Assess credible failures by examining the use of the DHI (process workflow) and the deviation from that use (within the process workflow). Document the findings in the form of a hazard table. Causes, deviations, or failures leading to hazards and hazardous situations are summarised as the following: Loss of function, Partial Loss of Function, Function provided when not required and Incorrect Function. The Hazard Effect must also include HSCs^1^ that highlight the problem or challenge should the hazard occur. The objective being to provide establish a connection between hazard and HSC for any given DHI class. See Tables 7–9 for the Hazard Analysis summary. Each of the hazards are also identified numerically in the care pathway (Figure 5).

**Table 7.** Hazard analysis summary.

ID	Hazard	Hazardous situation	Harm	Effect	
1	Hosting Infrastructure	Loss of function – User may not have access to the app features, e.g. test ordering, symptom check, advice and guidance. Inability to install the app from the app store.	Patient may be asymptomatic and come into contact with other people whilst testing positive for COVID-19. Delay in diagnosis and treatment.	Patient does not receive the current health status. Patient may have or be at risk of having COVID-19. Information: Delayed reporting of events. Lack of quality/reliable data. Insufficient utilisation of data and information. Quality: Poor patient experience Insufficient continuity of care	Utilisation: Low demand for services. Low adherence to treatments. Efficiency: Poor planning and coordination. Delayed provision of care. Cost: High cost of manual processes. Accountability: Absence of community feedback mechanisms. Inadequate understanding of beneficiary populations.
		Partial loss of function – User may have intermittent access to the app features, e.g., test ordering, symptom check, advice and guidance.			
		Incorrect Function – incorrect alerting, warning, or symptom status.	Patient receives an incorrect health status. Patient suffers unnecessary stress or anxiety due to incorrect health status.		
		Function provided when not required – App provides increased periodic alerting, warning, or symptom status than necessary.	Patient may become unreceptive to alerts and warnings – Alert Fatigue.	Patient may become unreceptive to alerts and warnings – Alert Fatigue. Information: Insufficient utilisation of data and information.	Quality: Poor patient experience

**Table 8.** Hazard analysis summary (continued).

ID	Hazard	Hazardous situation	Harm	Effect	
2	Symptom check/triage functionality	Loss of Function – incorrect clinical decision-supporting function.	Incorrect health status provided. Delay in diagnosis and treatment. Failure to isolate.	Patient does not receive the current health status. Patient may have or be at risk of having COVID-19. Information: Delayed reporting of events. Lack of quality/reliable data. Insufficient utilisation of data and information.	Quality: Poor patient experience Insufficient continuity of care Efficiency: Poor planning and coordination. Delayed provision of care. Cost: High cost of manual processes. Accountability: Insufficient patient engagement.
		Partial Loss of Function – intermittent loss of clinical decision-supporting functionality.			
		Function provided when not required – App provides increased periodic symptom status than necessary.	Patient may become unreceptive to alerts and warnings – Alert Fatigue.		
		Incorrect Function – incorrect alerting, warning, or symptom status.	Patient receives an incorrect health status. Patient suffers unnecessary stress or anxiety due to incorrect health status.		
3	Contact tracing (Bluetooth)	Loss of Function – failure to detect contacts using the Bluetooth functionality.	Patient may be asymptomatic and come into contact with other people whilst testing positive for COVID-19. Delay in diagnosis and treatment.	Patient does not receive the current health status. Patient may have or be at risk of having COVID-19. Information: Delayed reporting of events. Lack of quality/reliable data. Insufficient utilisation of data and information. Quality: Poor patient experience Insufficient continuity of care	Utilisation: Low demand for services. Low adherence to treatments. Efficiency: Poor planning and coordination. Delayed provision of care. Cost: High cost of manual processes. Accountability: Absence of community feedback mechanisms. Inadequate understanding of beneficiary populations.
		Partial Loss of Function – intermittent Bluetooth operation.			
		Incorrect function – incorrect alert and warning, including isolation advice provided.	Patient receives an incorrect health status. Patient suffers unnecessary stress or anxiety due to incorrect health status.		
4	Risk score of contact (contact tracing)	Loss of function – incorrect clinical decision-supporting function.	Incorrect health status provided. Delay in diagnosis and treatment. Failure to isolate.	Patient does not receive the current health status. Patient may have or be at risk of having COVID-19. Information: Delayed reporting of events. Lack of quality/reliable data. Insufficient utilisation of data and information.	Quality: Poor patient experience Insufficient continuity of care Efficiency: Poor planning and coordination. Delayed provision of care. Cost: High cost of manual processes. Accountability: Insufficient patient engagement.
		Partial Loss of Function – intermittent loss of clinical decision-supporting functionality.			
		Function provided when not required – App provides increased periodic symptom status than necessary.	Patient may become unreceptive to alerts and warnings – Alert Fatigue.		
		Incorrect Function – incorrect alerting, warning, or symptom status.	Patient receives an incorrect health status. Patient suffers unnecessary stress or anxiety due to incorrect health status.		

**Table 9.** Hazard analysis summary (continued).

ID	Hazard	Hazardous situation	Harm	Effect	
5	Test & Trace Service	Loss of Function – test and trace system outage nationally or regionally.	Patient receives an incorrect health status. Patient suffers unnecessary stress or anxiety due to incorrect health status. Patient may be asymptomatic and come into contact with other people whilst testing positive for COVID-19. Delay in diagnosis and treatment.	Patient does not receive the current health status. Patient may have or be at risk of having COVID-19. Information: Delayed reporting of events. Lack of quality/reliable data. Insufficient utilisation of data and information. Quality: Poor patient experience. Insufficient continuity of care.	Efficiency: Poor planning and coordination. Delayed provision of care. Cost: High cost of manual processes. Accountability: Insufficient patient engagement.
		Partial Loss of Function – inefficient provision of test status to users.			
		Function provided when not required – incorrect function.			
6	Accessibility/Useability User Interface	Loss of Function – software does not function correctly to required use case or user flow.	Patient receives an incorrect health status. Patient suffers unnecessary stress or anxiety due to incorrect health status. Patient may be asymptomatic and come into contact with other people whilst testing positive for COVID-19. Delay in diagnosis and treatment.	Patient does not receive the current health status. Patient may have or be at risk of having COVID-19. Information: Delayed reporting of events. Lack of quality/reliable data. Insufficient utilisation of data and information.	Quality: Poor patient experience. Insufficient continuity of care. Efficiency: Poor planning and coordination. Delayed provision of care. Cost: High cost of manual processes. Accountability: Insufficient patient engagement.
		Partial Loss of Function – software does not function correctly to required use case or user flow.			
		Function provided when not required – software does not function correctly to required use case or user flow.			
		Incorrect Function – software does not function correctly to required use case or user flow.			

### Step four – examination of safety significance & control

Examine safety significance of each hazard and identify safety controls. This application of CRM methodology informs the hazard analysis. Examine DHI hazard(s) using likelihood (L) and severity (S) to derive a severity rating (R – safety significance). Identify safety controls that enable mitigation of hazards identified, Tables 10–12 provides the summary results of the examination of causes, controls, initial and residual hazard assessment.

**Table 10.** Safety controls.

ID	Possible causes	Existing controls	Initial assessment L × S = C	Residual Controls	Residual Assessment L × S = C	Justification
1	Hardware component failure. Software component failure. App store unavailable. Service capacity limits exceeded.	None – the introduction of a new DHI to provide mobile health services aligned to – Targeted client communication (targeted communication to individuals)	L × M = 3	Hosted services must provide resilience, capacity, capability, failover, backup and meet the intended performance requirements of the DHI. Manufacturer to client service contract must ensure the provision of support for any failure to meet intended operating claims. Software quality assurance activities should provide evidence to support the specification of the DHI for both functional and non-functional operation. Periodic assurance activities must provide evidence to support meeting original specifications over the lifetime the DHI is in use.	VL × M = 2	Hosted services provide a high availability infrastructure with near constant monitoring for service performance. Service availability can be provided at scale within a strict contractual environment.
2	User enters data incorrectly. Symptom checking software component failure. Incorrect symptom check configuration. Inaccurate efficacy of clinical decision-support functionality.	None – the introduction of a new DHI to provide mobile health services aligned to – Targeted client communication (targeted communication to individuals)	L × M = 3	Clinical decision-supporting functionality must be designed and maintained with the appropriate clinical subject matter expertise and governance, throughout the lifetime of the DHI intended operation. All changes and modifications to symptom checking functions must be assessed and assured by appropriately skilled health-care professionals. All clinical decision-supporting features must align to useability requirements for the appropriate intended user – e.g. reading comprehension, languages and accessibility requirements.	VL × M = 2	Clinical decision-supporting functionality developed with healthcare professional supervision and curation provides a more robust claim and evidence to support safer and correct operation. Gamification of symptom checking applications can occur, however operating as intended does depend on correct data input by the user under normal conditions.

**Table 11.** Safety controls (continued).

ID	Possible causes	Existing controls	Initial assessment L × S = C	Residual controls	Residual assessment L × S = C	Justification
3	No Bluetooth signal – functionality switched off. Self-isolation breach. Hardware component failure – smartphone replacement. Useability/interoperability failure – multiple phones used by one user.	None – the introduction of a new DHI to provide mobile health services aligned to – Targeted client communication (targeted communication to individuals)	M × M = 3	The dependency on third-party APIs and hardware configurations that control the near field communication properties of the DHI must be dependable. The user must be warned of the status of the Bluetooth settings and how to manage the configuration.	L × M = 3	The DHI's intended purpose is best enabled when contact tracing functionality is enabled. The DHI provides symptom guidance, simple CDS functionality and BLE tracing via third party APIs. If the configuration is maintained and the user knows that contact tracing functionality is unavailable if BLE is switched off, there is sufficient instruction for safe operation provided.
4	User enters data incorrectly. Encounter detection error. Hardware component failure – App unavailable. Useability error – user wearing PPE during contact scenarios. Hardware component error – no Bluetooth signal (functionality switched off).	None – the introduction of a new DHI to provide mobile health services aligned to – Targeted client communication (targeted communication to individuals)	L × M = 3	Clinical decision-supporting functionality must be designed and maintained with the appropriate clinical subject matter expertise and governance, throughout the lifetime of the DHI intended operation. All changes and modifications to risk scoring functions must be assessed and assured by appropriately skilled health-care professionals. All clinical decision-supporting features must align to useability requirements for the appropriate intended user – e.g. reading comprehension, languages and accessibility requirements.	VL × M = 2	Clinical decision-supporting functionality developed with healthcare professional supervision and curation provides a more robust claim and evidence to support safer and correct operation. Gamification of symptom checking applications can occur, however operating as intended does depend on correct data input by the user under normal conditions.

**Table 12.** Safety controls (continued).

ID	Possible causes	Existing controls	Initial assessment L × S = C	Residual controls	Residual assessment L × S = C	Justification
5	Hardware component failure. Software component failure. User enters data incorrectly. Service capacity limits exceeded (test and trace service).	None – the introduction of a new DHI to provide mobile health services aligned to – Targeted client communication (targeted communication to individuals)	L × M = 3	Hosted services must provide resilience, capacity, capability, failover, backup and meet the intended performance requirements of the DHI. Manufacturer to client service contract must ensure the provision of support for any failure to meet intended operating claims. Software quality assurance activities should provide evidence to support the specification of the DHI for both functional and non-functional operation. Periodic assurance activities must provide evidence to support meeting original specifications over the lifetime the DHI is in use. External controls and mitigation are dependent upon the service provided of test and trace. The required 24hr service provision should be monitored closely as a key part of the response to COVID-19 and pandemic management response requirements. Additional guidance should be provided in the event of a delay in reporting test status.	VL × M = 2	A critical requirement of the test and trace service is the provision of results within 24hrs. Capacity and demand are key enablers to hazard mitigation. Government health policy and guidance in the event of any failure to provide a resilient service should take into consideration the risk factors and patient safety issues that may result.
6	User enters data incorrectly. Encounter detection error. Hardware component failure – App unavailable. Useability error – user wearing PPE during contact scenarios. Hardware component error -no Bluetooth signal (functionality switched off).	None – the introduction of a new DHI to provide mobile health services aligned to – Targeted client communication (targeted communication to individuals)	M × C = 3	The development of the DHI aligned to useability and accessibility standards, including independent assurance of meeting such requirements, will provide control. The use of selectable languages for a full spectrum of public engagement including the appropriate clinical governance and public health advice on the structure, format and content of advice provided would further add to the mitigation.	L × C = 2	Compliance with recognised standards in this area and good practise in content change management should provide sufficient control to this hazard.

### Example 2 – remote patient monitoring

Delivering care to patients within the comfort and safety of their own homes (including care homes) is being enabled by the UK NHS through the launch of the remote monitoring programme.^54^ This example considers a remote patient monitoring solution implemented across a region in the UK. The system enables the regular remote capture of patient-reported outcome measures (PROMs) between appointments to support shared decision-making about when patients needed care the most. The solution aligns to a UK government policy and strategy for increasing the scale and capability of digitally enabled care in the home.

### Step one – establish the digital health intervention functional description (DHI selection)

Select the functional description from Table 3 that represents the DHI, including the definition, synonyms and other descriptors. Table 13 summarises the functional description for this example DHI.

**Table 13.** DHI functional description.

Digital health intervention	
Client-to-provider telemedicine	
Definition	Synonyms and Other Descriptors
Provision of health services at a distance; delivery of health services where clients/patients and healthcare workers are separated by distance	Consultations between remote client/ patient and healthcare worker Clients/patients transmit medical data (e.g. images, notes videos) to healthcare worker

### Step two – analyse the DHI-enabled care pathway

**Figure 6.**
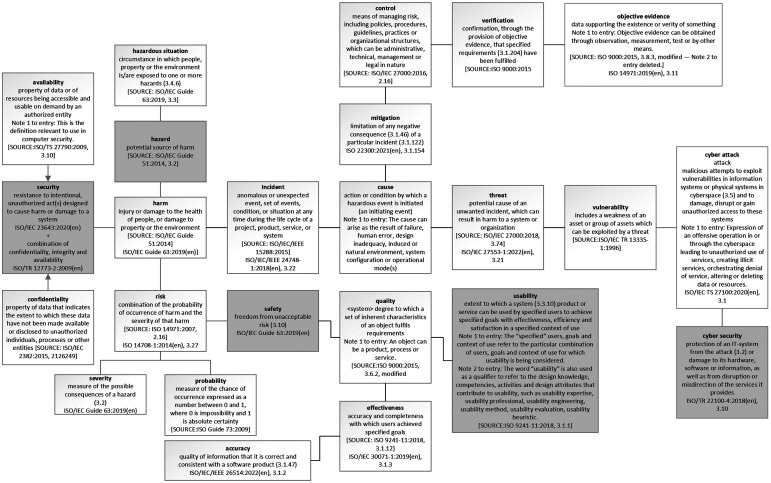
Contributing risk factors conceptual model.

Document the care pathway that the DHI is used (Figure 6). Ensure each DHI example has the required minimum evidence for analysis and assessment – product name, workflow (flowchart), explanation of intended use, technical operation and features or functions (clinical speciality). Table 14 provides the summary for this example DHI.

**Table 14.** DHI product name and technical operation summary evidence.

Product name	Remote patient monitoring application
User Type or Role	Anyone aged 16 or over who lives in England or Wales can use the remote patient monitoring application. Parents and guardians and those patients under 16 are also within scope (within consent guidelines).
DHI technical operation	The DHI is an application running multiple remote patient monitoring services on one platform. The application conforms to the UK NHS interoperability and industry integration requirements for the portability of patient data and onward decision-making needs. Collection of patient data is achieved via a method of the patient's choice within a virtual ward setting, allowing data to be shared directly with the healthcare professional responsible for treatment. All data is automatically updated onto the patient's electronic medical record.
DHI features or functions	Patients take more active roles in their care. Increase in digital inclusion for all communities. Healthcare professionals can remotely monitor patients. Healthcare professionals can save time and more efficiently manage patient lists through telehealth functionality.
Other Information	Patient portal access.
Care Pathway	See Figure 6

### Step three – perform hazard identification

Assess credible failures by examining the use of the DHI (process workflow) and the deviation from that use (within the process workflow). Document the findings in the form of a hazard table. See Table 15 for the Hazard Analysis. Each of the hazards are also identified numerically in the care pathway (Figure 6).

**Table 15.** Hazard analysis summary.

ID	Hazard	Hazardous situation	Harm	Effect	
1	DHI failures	Loss of DHI functionality – full or partial (intermittent) failure to operate as intended.	Patient receives incorrect diagnosis. The user would not be able to complete the data entry or other key functions which could result in a delay in processing key information or an inconvenience to the patient.	Patient does not receive the current health status. Information: Delayed reporting of events. Lack of quality/reliable data. Insufficient utilisation of data and information. Inadequate supportive supervision. Efficiency: Poor planning and coordination. Delayed provision of care. Quality: Poor patient experience Insufficient continuity of care	Utilisation: Low demand for services. Low adherence to treatments. Cost: High cost of manual processes. Accountability: Absence of community feedback mechanisms. Inadequate understanding of beneficiary populations.
		System(s) failure, Hardware, or Network issues – e.g., bandwidth, signal strength, including intermittent problems.			
		Incorrect presentation of clinical data.			
2	Use of DHI	Incomplete or incorrect on-boarding of the technology or implementation of the DHI.	Delay in the diagnosis and ongoing care of the patient. Patient deterioration.	Patient does not receive the current health status. Information: Delayed reporting of events. Lack of quality/reliable data. Insufficient utilisation of data and information. Efficiency: Poor planning and coordination. Delayed provision of care.	Quality: Poor patient experience Insufficient continuity of care Cost: High cost of manual processes. Accountability: Insufficient patient engagement.
		Inadequate workflow management.			
		Poor planning and coordination.			
3	Useability or Human Factors issues within the use of the DHI	Key Patient and/or User information communication issues, barriers or roadblocks including limited access to key information.	Delay in the diagnosis and ongoing care of the patient. Patient deterioration.	Patient does not receive the current health status. Information: Delayed reporting of events. Lack of quality/reliable data. Insufficient utilisation of data and information. Quality: Poor patient experience Insufficient continuity of care	Efficiency: Poor planning and coordination. Delayed provision of care. Cost: High cost of manual processes. Accountability: Absence of community feedback mechanisms. Inadequate understanding of beneficiary populations. Utilisation: Low demand for services. Low adherence to treatments.
4	Incorrect Clinical Diagnosis	Incorrect patient data uploaded	Patient receives incorrect diagnosis. Delay in the diagnosis and ongoing care of the patient. Patient deterioration.	Patient does not receive the current health status. Information: Delayed reporting of events. Lack of quality/reliable data. Insufficient utilisation of data and information. Quality: Poor patient experience. Insufficient continuity of care.	Efficiency: Poor planning and coordination. Delayed provision of care. Cost: High cost of manual processes. Accountability: Insufficient patient engagement.
		Clinical user error on data entry			
		Incorrect treatment chosen			
		Insufficient health and care worker competence			

### Step four – examination of safety significance & control

Examine safety significance of each hazard and identify safety controls, Tables 16 and 17 provides the summary results of the examination of causes, controls, initial and residual hazard assessment.

**Table 16.** Safety controls.

ID	Possible causes	Existing controls	Initial assessment L × S = C	Residual controls	Residual assessment L × S = C	Justification
1	Hardware component failure. Software component failure. Service capacity limits exceeded.	None – the introduction of a new DHI to provide remote patient health services aligned to – CLIENT-TO-PROVIDER TELEMEDICINE	M × C = 3	Hosted services must provide resilience, capacity, capability, failover, backup and meet the intended performance requirements of the DHI. Manufacturer to client service contract must ensure the provision of support for any failure to meet intended operating claims. Software quality assurance activities should provide evidence to support the specification of the DHI for both functional and non-functional operation. Periodic assurance activities must provide evidence to support meeting original specifications over the lifetime the DHI is in use.	VL × C = 2	Hosted services provide a high availability infrastructure with near constant monitoring for service performance. Service availability can be provided at scale within a strict contractual environment.
2	Monitoring of the implementation is not in place or is incorrectly assessed as successful. Incorrect user details entered for notification or registration with the DHI. Incorrect timing and scheduling of patient requests for treatment.	None – the introduction of a new DHI to provide remote patient health services aligned to – CLIENT-TO-PROVIDER TELEMEDICINE	VL × M = 2	It is very unlikely that any DHI is implemented without regard to the impact and change in the patient pathway, Standard Operating Procedures, or similar.	VL × M = 2	Integration of this product into existing workflows and the correct data landing in patient records is key. Further mitigation will be sought in this area from other implementations (best practice) and local user setup and training.

**Table 17.** Safety Controls (continued).

ID	Possible causes	Existing controls	Initial assessment L × S = C	Residual controls	Residual assessment L × S = C	Justification
3	Useability barriers preventing important information critical to the care of the patient being communicated and/or lack of access to information or data. Human Error- Incorrect email address entered for notification; unauthorised person receives results. Lack of patient usage or reporting within the system (digital literacy issues).	None – the introduction of a new DHI to provide remote patient health services aligned to – CLIENT-TO-PROVIDER TELEMEDICINE	M × C = 3	Patient engagement is always key to measure the effectiveness and outcomes of any DHI. In this instance, patient engagement is critical due to the nature of the remote patient care required. Additional diligence and collaboration with the patient should ensure that this is unlikely to impact the care provided.	VL × C = 2	Human Factors errors are prevalent in all DHIs. As such it is an organisations duty to proactively monitor the quality of care provided using such tools to support the health and care of patients. As a product that is implemented elsewhere in the UK, it is assumed that a large proportion of human factors issues have been designed out. Training that is effective will provide additional mitigation and control.
4	Poor adherence to the guidelines and standard operating procedures. Lack of or inappropriate referrals. Clinical tasks are not updated or incorrectly received (through data error or user error). Patient registration issues within the health organisation. Inadequate or inefficient training on the new digital health implementation provided.	None – the introduction of a new DHI to provide remote patient health services aligned to – CLIENT-TO-PROVIDER TELEMEDICINE	M × C = 3	There is a dependency on the timeliness of data reporting and transmission across the system. The impact of any failures in this regard is considerable and could be more likely to occur if the DHI is not implemented with careful consideration to the care pathway in which it is intended to be used.	VL × C = 2	There is a dependency on the timeliness of data reporting and transmission across the system. The impact of any failures in this regard is considerable and could be more likely to occur if the mitigation and controls suggested are not actioned. Health and care professionals have a duty of care with their patients. This does provide some mitigation, but not complete if the implementation and use is not diligently operated. It is very unlikely that users will not receive sufficient training with the DHI to provide their role.

## Results summary

A qualitative analysis of results was completed based on the authors’ experience. Application of the method used, provided the hazard assessment and the results used to generate an assurance case, thereby providing a justification of safety and eliciting confidence that the DHI is fit for purpose. Avoidance of hazards through mitigating controls contributes to meeting the HSC. From the subject matter experience and suitably qualified personnel performing the HazID and assessment, we can see the impact on patient safety can be defined by either a delay in care or diagnosis or inappropriate care or diagnosis provided. These provide two useful terms that can be used in a more structured context for harm events. The method provides indication that by structuring the hazard assessment this way, including the DHI classes, together with HSCs; ontologies improve the quality of assessment. This is achieved through the use of consistent terminology and enables repeatable assessments to be completed with similar DHIs. Considering the impact on patient safety for a DHI could be misdiagnosis or delay in diagnosis; the impact would naturally be a delay or inappropriate treatment provided. Whilst we acknowledge the individual domains that contribute to the safety of a DHI, the focus of this research is the overall quality of safety claim within the regulatory challenges of compliance or certification and not any single domain such as human factors, useability, security, or other contributory risk factors. The combined contribution is key and underpinned by the flexibility of risk methodology created within this research. We postulate through our method, that improvements in the categorisation, use of consistent language and terminology allows for a broader inclusion of contributory factors and mixed method risk assessments due to the efficiency in risk analysis undertaken.

## Discussion

### Contributory risk factors

The regulations and standards, including country specific legislation for DHI product safety includes the contributing risk factors of safety, security and useability. Security encompasses cyber security and privacy. Risk analysis methodologies applied within this methodology must incorporate all risk factors that impact the safety of the DHI. Disparate risk assessments have the disadvantage of lower quality safety analysis through a lack of consideration to all contributing risk factors (unstructured safety claims). We acknowledge the contribution that more specialised risk analysis methods bring, specifically to IoMT DHIs, useability engineering, cyber security threat analysis, for example.^17,21^ With the addition of the classification scheme and HSCs, we provide a hazard assessment that incorporates sociotechnical challenges in a structured and simplified format. When this information is incorporated into the hazard analysis from a conceptual modelling perspective, it is clear to see how each risk factor may contribute to the overall safety claim being made (structured approach). We can see from the conceptual models (Figure 6), that safety claims are dependent on many risk factors and if structured correctly, can provide the essential evidence to support DHI compliance to regulatory requirements.

From a hazard and risk analysis perspective, the methodology allows for the categorisation of risk to contributory risk factors (Table 18) and a direct relationship to DHI classification and HSC.

**Table 18.** Examples of categories of DHI hazards.

Hazard category	Examples of categories
Useability ^55–57^	Wrong entry/retrieval Did not enter/retrieve Partial entry/retrieval Delayed entry/retrieval
Security ^15,58^	Confidentiality Integrity Availability
Functionality ^55–57^	Interface with third-party systems Software functionality Incorrect input Incorrect output System configuration Software not accessible

### Structured safety claims

**Figure 7.**
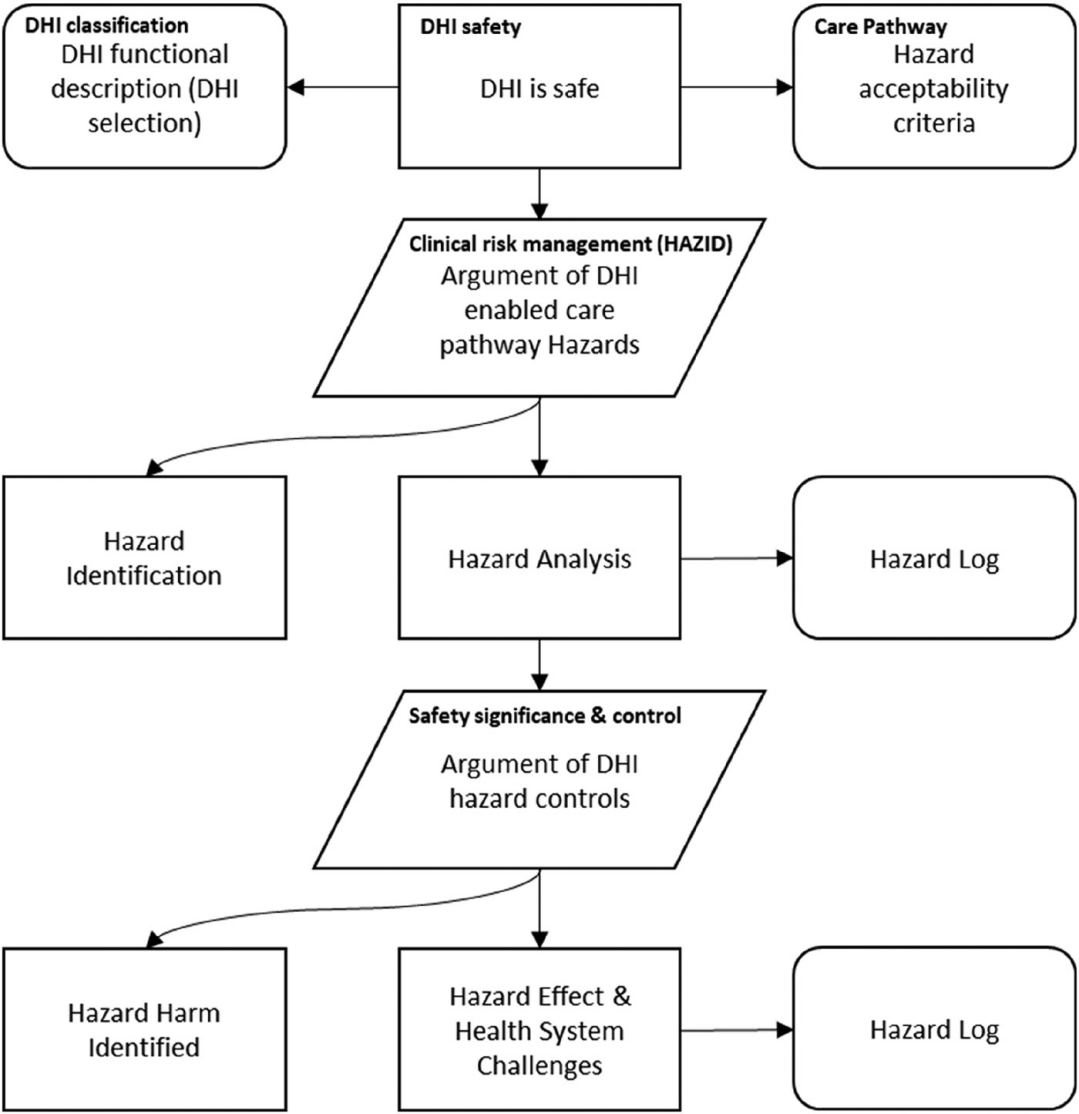
GSN safety argument for DHI.

The safety claim provides an argument that a system is acceptably safe for the intended operation and in a digital health context, intended use. These arguments are commonly represented visually in medical devices industries using Goal Structuring Notation (GSN).^59–61^ The example DHIs assessed using the method, are developed into structured safety claims (Figure 7), that represent the initial HazID and assessment completed.

The hazard summary tables provide detailed uniquely identified hazards, hazardous situations, harm, effect and further risk control parameters. The GSN arguments highlight the claims made that illicit confidence in the DHI safety through the method applied. Each step in the method and safety claim is supported by evidence in the context of the argument made. The objective of the safety claim and supporting evidence is to provide a compelling and comprehensive case for the safety of a DHI. Inadequately defined evidence will lead to false conclusions. Knowledge gaps has the potential for assurance deficits and directly impact the confidence in claim being made. The quality improvements enabled by the DHAM method are as a result of structure, terminology and clarity of content provided (i.e., ontologies) of the hazard assessment made.

### Health system challenges

HSCs provide a terminology that describes hazardous situations derived from the hazards identified. This allows for ontologies to be used for the structuring of hazard assessments. From a CRM perspective, we often see variations in quality of the safety claim with limited context on patient safety harms and effects.^10^ Hazard controls have been documented in detail to summarise and to aid the CRM and risk acceptability process. However, we can categorise these controls or group them into areas such as design, process, technical assurance, training, external design and external process. These groupings allow for improvements in risk acceptability, assessing and examining areas where standard termed controls are applied and the completeness of those controls documented. Safety critical industries often utilise database structures^61^ to further improve the recording of hazards and provision of a library of terms to prevent duplication of effort during HazID on subsequent systems (new or enhanced) with similar contexts, intended uses or use cases. We postulate that DHI hazard assessment and analysis can be completed more efficiently and to increased quality using structured HazID methods, thus removing ambiguity or subjectivity of claims made.

## Conclusion

The method provides indication that by structuring the hazard assessment this way, including the DHI classes, together with HSCs; ontologies improve the quality of assessment. This is achieved through the use of consistent terminology and enables repeatable assessments to be completed with similar DHIs. Considering the impact on patient safety for a DHI could be misdiagnosis or delay in diagnosis; the impact would naturally be a delay or inappropriate treatment provided. This suggests the impact on patient safety can be defined in a structured way, for example:

- patient receives incorrect diagnosis
- delay in the diagnosis and ongoing care of the patient
- patient deterioration
- patient receives an incorrect health status
- patient suffers unnecessary stress or anxiety due to incorrect health status.

Hazard tables or hazard logs, present clarity and concise summaries that support CRM activities already discussed during this method. This method can be applied to many more DHIs leading to further work to assess the contribution of improvements in the quality of assessment of DHIs as a case study, beyond the two examples used in this research. Digital Health regulatory and standards compliance require risk or hazard assessments to be made available for inspection or assessment by those third parties providing certification. This allows us to identify hazards using a more defined terminology and ease regulatory burdens. Although we utilise a HazID methodology, other methods are available and all have their advantages and disadvantages when applied during the various stages of a development lifecycle. The choice of methodology is not under analysis, more the articulation of the assessment results to support any claims made.

There is a dependency on this information to inform the reader to perform various roles – regulatory certification, risk acceptance, implementation and the use of DHI. The use of ontologies aids initial hazard assessments by improving the dissemination of information from different subject areas or contexts and removing communication barriers between individual domain experts (e.g. clinical, technical). This exhibits the similar objectives of communication improvement within the original WHO DHI classification guidance. The quality of initial hazard assessment increases and provides greater insight into the hazardous situations and overall hazard effect. For a DHI to be implemented and used within the health industry, applicable regulatory and standards compliance activities are required. The effort to comply with these regulations and standards is great and often seen as a barrier to digital health innovations. Therefore, standardising the approach of HazID, assessment and analysis in this method and documenting the hazard summary using ontologies such as HSC and categories of controls offers a solution to this problem.

### Further work

Further work will examine the qualitative aspects of this method for other DHIs. Additional DHIs assessed will provide the quantitative view. This is intended to assist with creating the ontology and terminology required and the structured language in which to improve the communication of hazards to stakeholders. We intend to apply Model Driven Engineering & object-oriented methodology, to derive these ontologies. Using the conceptual model as a representation of the problem domain. Using concepts, associations and attributes to form part of an overall model, this can be used to interpret the problem. Concepts have categories – physical, specifications, places etc. And an association is a relationship between concepts and indicates a connection between these concepts.

The objective by using model driven engineering and object-oriented methodologies is to investigate and examine the terminologies and concepts used to explore their relationship for further analysis. UML notation format provides a static structural diagram that represents the problem. By following the steps below, we aim to validate the method, the model, metamodel and instances developed.

1. Analyse and identify the key elements of the problem (system), the conceptual classes within the problem
2. Identify the properties and relationships between these elements
3. Identify any constraints (limitations) and conditions of the model
4. The resultant conceptual model is represented for peer review as a class diagram; and finally
5. Examples (instances) are developed from the conceptual model to validate the metamodel created – object diagram.

Further work is needed, to demonstrate this method across the full DHI classification scheme, providing quantitative analysis and results. The implementation and verification of DHIs, justified this way, provide a direct correlation to the HSC. In turn, through the development of ontologies we can further validate the methodology utilising other risk methodologies including mixed method approaches. The objective being to strengthen safety claims and provide direct correlation to HSCs.
